# *LowDoseWizard*: rapid and standardized setup of low-dose cryo-TEM imaging in *SerialEM*

**DOI:** 10.1107/S2059798326006327

**Published:** 2026-07-28

**Authors:** Simon A. Fromm, Simone Mattei

**Affiliations:** a EMBL Imaging Centre European Molecular Biology Laboratory Meyerhofstrasse 1 69117Heidelberg Germany; b Molecular Systems Biology Unit European Molecular Biology Laboratory Meyerhofstrasse 1 69117Heidelberg Germany; National Center of Biotechnology, CSIC, Spain

**Keywords:** low-dose imaging, *SerialEM*, cryo-EM, cryo-ET

## Abstract

Structure elucidation of biological macromolecules by single-particle cryogenic electron microscopy (SPA cryo-EM) or cryogenic electron tomography (cryo-ET) relies on low-dose imaging on cryogenic transmission electron microscopes (cryo-TEMs). Routine microscope setup remains technically demanding and can be time-consuming, particularly for inexperienced or infrequent users. We present *LowDoseWizard*, a guided workflow implemented in *SerialEM* that enables rapid and standardized setup of cryo-TEM imaging conditions. From minimal user input, the workflow configures microscope optics, camera parameters and image-shift settings for all low-dose states, and guides the user through key daily alignment procedures, including beam-shift offset calibration, objective-lens astigmatism correction and coma-free alignment. The workflow is organized into modular routines that can be executed sequentially or independently, while microscope-specific acquisition parameters are defined in editable configuration files, allowing flexible adaptation to different instruments without modification of the core scripts. Across user sessions on three microscopes at EMBL Heidelberg, the complete setup required on average less than 15 min. To assess whether predefined imaging conditions generated by the workflow are compatible with high-resolution data collection, we acquired apoferritin data on a 200 kV Glacios and a 300 kV Titan Krios. These datasets yielded reconstructions at 1.62 and 1.09 Å resolution, respectively, demonstrating that rapid, guided setup can support near-atomic and atomic-resolution SPA cryo-EM. *LowDoseWizard* lowers the barrier to robust cryo-TEM setup, reduces the time spent on routine parameter selection and alignment, and helps users focus on sample-specific aspects of data acquisition such as target selection. The workflow should be particularly valuable in shared instrumentation environments, where accessibility, reproducibility and efficient microscope use are critical.

## Abstract 2

*LowDoseWizard* is a guided *SerialEM* workflow for rapid and standardized setup of low-dose cryo-TEM imaging conditions for single-particle analysis and cryo-electron tomography.

## 1. Introduction

Cryo-EM has become a central method for the structural analysis of biological macromolecules by single-particle analysis and for the investigation of molecular organization *in situ* by cryo-ET. This progress has been driven by major advances in instrumentation, including the introduction of direct electron detectors (DEDs) with improved detective quantum efficiency (DQE) and readout speed (Campbell *et al.*, 2012 ▸; Li *et al.*, 2013 ▸; Bai *et al.*, 2013 ▸; McMullan *et al.*, 2014 ▸; Ruskin *et al.*, 2013 ▸; Peng *et al.*, 2023 ▸), and the implementation of high-throughput acquisition strategies such as fringe-free imaging (FFI) and aberration-free image shift (AFIS) (Cheng *et al.*, 2018 ▸; Eisenstein *et al.*, 2023 ▸, 2024 ▸). In parallel, developments in image processing have made both single-particle cryo-EM (Scheres, 2012 ▸; Punjani *et al.*, 2017 ▸; Kimanius *et al.*, 2016 ▸) and cryo-ET (Tegunov & Cramer, 2019 ▸; Tegunov *et al.*, 2021 ▸; Zivanov *et al.*, 2022 ▸; Burt *et al.*, 2024 ▸) increasingly robust and widely applicable. As a result, modern cryo-TEM workflows can generate very large quantities of high-quality data, placing increasing importance on acquisition procedures that are reliable, reproducible and matched to the specific experimental goal.

Despite these advances and the availability of automated sample-screening and data-collection software (Bouvette *et al.*, 2022 ▸; Cheng *et al.*, 2023 ▸), the routine setup of low-dose imaging conditions on high-end cryogenic transmission electron microscopes remains technically demanding. The choice of beam conditions, magnification, fluence, defocus, camera parameters and alignment procedures depends on both the microscope configuration and the intended application, whether screening, single-particle analysis (SPA) or tomography. This challenge is particularly relevant in shared facilities, where users may have widely differing levels of experience and may spend only limited time at the microscope relative to sample preparation and downstream data processing. Commercial acquisition packages provide guided environments for routine operation, but may offer limited flexibility when workflows need to be adapted to specific specimens or when new acquisition strategies are to be implemented. By contrast, open-source software such as *SerialEM* (Mastronarde, 2005 ▸) provides extensive control over microscope operation and has become a widely used platform for the development and application of advanced cryo-EM and cryo-ET workflows. Nevertheless, *SerialEM*’s lack of guided workflows and its open design allowing maximal flexibility and full control of nearly all imaging parameters also requires greater user expertise during setup.

To make low-dose cryo-TEM setup faster, more reproducible and more accessible without sacrificing the flexibility of *SerialEM*, we developed *LowDoseWizard*, a guided workflow for configuring low-dose imaging sessions from minimal user input. The workflow automatically defines beam parameters, magnifications and camera settings for different imaging tasks and guides the user through key daily calibration and tuning steps. Microscope-specific settings are stored in editable configuration files, allowing adaptation to different instruments while preserving a common workflow structure. Here, we describe the design and implementation of *LowDoseWizard* and evaluate its use in routine cryo-TEM setup. We show that the workflow enables complete setup within approximately 15 min and that the resulting predefined imaging conditions support high-resolution single-particle cryo-EM data collection, as demonstrated by apoferritin reconstructions at 1.62 Å resolution on a Glacios and 1.09 Å resolution on a Titan Krios.

## 2. *LowDoseWizard* design and implementation

### 2.1. Workflow overview

**Figure 1:**
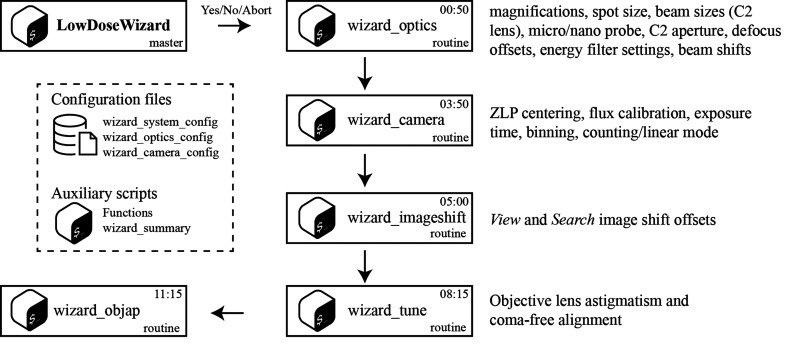
*LowDoseWizard* workflow. The times specified on the top right indicate the respective start time of that routine in Supplementary Video S1.

*LowDoseWizard* organizes cryo-TEM low-dose setup into a sequence of modular routines that can be executed consecutively or independently, depending on the needs of the session. The workflow is typically initiated after acquisition of a grid map (also termed an ‘atlas’) and proceeds from configuration of microscope optics to setting of camera parameters, followed by calibration of image-shift offsets and final tuning steps such as astigmatism correction and coma-free alignment (Fig. 1 ▸). All routines are launched from a single master script, and each routine begins with a limited set of user prompts relevant to the specific imaging task (Supplementary Video S1). From these inputs, the remaining parameters are determined automatically. Manual intervention via the TEM control hand panels is required only where direct microscope interaction is necessary, and these steps are accompanied by explicit prompts within the workflow.

**Figure 2:**
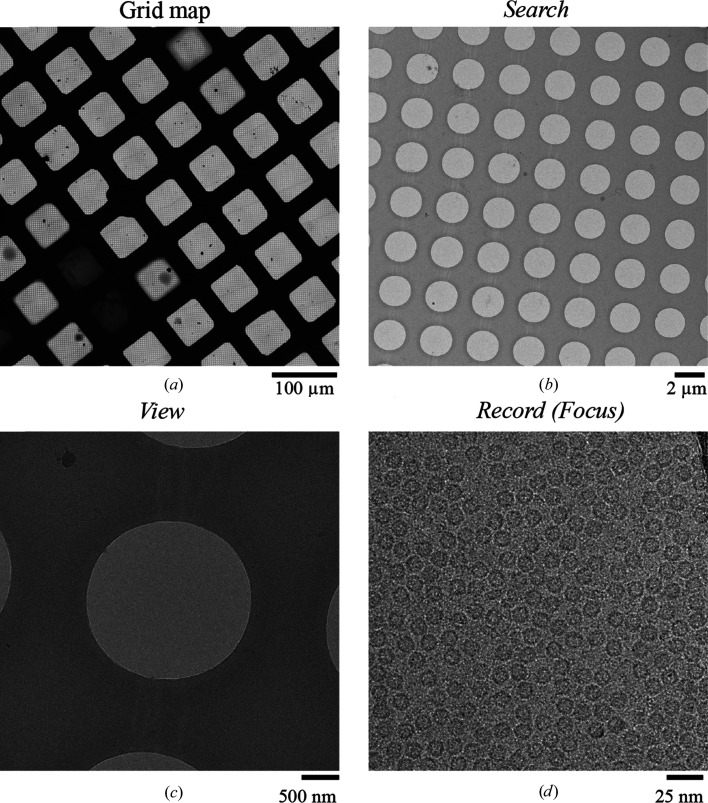
Low-dose states used in the *LowDoseWizard* workflow. Typical field of view of an (*a*) grid map (100× nominal magnification, calibrated pixel size 1280 Å), (*b*) *Search* (2250×, 56 Å), (*c*) *View* (11 500×, 11.2 Å) and (*d*) *Record* image (215 000×, 0.572 Å). Images are taken from the data-acquisition session on the Krios microscope.

The only prerequisite for execution is the definition in the *SerialEM Navigator* of one position over vacuum, labelled ‘empty’, and one position on a sacrificial specimen area, labelled ‘sample’. These positions provide reference locations for parameter setup and calibration. The workflow relies on the standard *Low Dose areas* defined in *SerialEM* (also referred to as states but not to be confused with *SerialEM Imaging States*; *SerialEM* terminology is depicted in italics). *Search* is used for mapping of grid squares or lamellae, *View* for refinement of the target position following the last stage movement, *Focus* for setting the desired image defocus and *Record* for data acquisition (Fig. 2 ▸). In tomography applications, *Trial* is kept identical to *Focus* for tracking purposes during tilt-series acquisition. The workflow was developed and tested on FEI/Thermo Fisher Scientific microscopes, and the microscope-specific terminology used throughout reflects this implementation context.

### 2.2. Automated configuration of imaging conditions

The first stage of the workflow, named ‘optics routine’, establishes the low-dose states. Because all subsequent steps depend on correctly configured *Low Dose areas*, this part of the setup has to be completed at the ‘empty’ position before calibration and tuning routines are performed. *LowDose­Wizard* distinguishes between tomography and single-particle workflows and adapts follow-up prompts accordingly. Depending on the selected application, the user specifies a small number of parameters, including the desired *Record* magnification, the required level of map detail and, for single-particle analysis, characteristics of the foil holes and support material. From these inputs, the workflow defines the appropriate settings for all *Low Dose areas*, including magnification, spot size, beam size (or C2 lens strength for two-condenser systems), C2 aperture size, minicondenser lens (microprobe or nanoprobe), defocus offsets and, where applicable, energy-filter slit width and offset. These settings are not hard-coded but are selected from microscope-specific definitions stored in a configuration file (wizard_optics_config.txt; Table 1 ▸). This allows expert users or microscope managers to establish instrument-appropriate presets once, while routine users interact only with the simplified front-end of the workflow.

The underlying parameter-selection logic is designed to preserve robust imaging conditions while adapting to different experimental goals. *Record* settings are determined directly from the user-selected magnification and the optics configuration file. Spot size is kept constant across *Low Dose areas* to minimize lens hysteresis during the session. For tomography on three-condenser systems, *View* is set to the lowest magnification that still provides a parallel beam in nanoprobe mode. For single-particle applications, the *View* magnification is defined to yield a field of view (FOV) of approximately twice the user-specified foil-hole diameter, although this factor can be adjusted in the configuration file. *Search* settings likewise depend on the imaging mode: for single-particle acquisition, the lowest suitable selected-area (SA) magnification is used, whereas for tomography the user can choose between minimal, medium and high detail preset levels for target selection. Where applicable, energy-filter settings are applied across all states, and for SPA sessions on gold foil *Search* can be configured for plasmon imaging (Hagen, 2022 ▸). At the end of the optics routine, the user is guided through beam-shift offset calibration to ensure a centred beam in all *Low Dose areas*.

The second stage, named ‘camera routine’, configures the camera parameters. Here, the user provides only the desired fluence per tilt for tomography or the desired *Record* fluence for SPA acquisition. The workflow then performs electron-flux calibration in the *Record* state at the ‘empty’ position and, if an energy filter is present, carries out zero-loss peak (ZLP) centring beforehand. From the calibrated flux, exposure times for all *Low Dose areas* are assigned automatically. As for the optics, these settings are defined through an editable camera configuration file (wizard_camera_config.txt, Table 1 ▸), which contains microscope-specific presets for quantities such as fluence, exposure time, binning and image processing. The only manual intervention required during this step is confirmation, and if necessary correction, of beam centring in the *Record* area before flux calibration is performed. Together, these two setup stages translate a limited number of user decisions into a complete set of low-dose imaging and camera parameters tailored to the intended acquisition task.

### 2.3. Calibration and tuning routines

After the low-dose states and camera conditions have been defined, *LowDoseWizard* performs the calibration and tuning routines required for data acquisition. Image-shift calibration is carried out by the ‘image-shift routine’ at the ‘sample’ position for the *View* and *Search* areas. Before the calibration step, the user may interactively refine the stage position to ensure that suitable image features are present in the field of view, for example contamination, foil-hole edges or cellular structures. After eucentric height has been established, image-shift offsets are calibrated automatically using the *SerialEM* command *FindLowDoseShiftOffset*. To guard against erroneous calibration, the resulting values are compared with predefined limits stored in the optics configuration file. If these limits are exceeded, the user is warned and can choose to repeat the calibration, keep the measured values or reset the offsets to zero.

Microscope tuning is implemented as a guided ‘tuning routine’ for objective-lens astigmatism correction and coma-free alignment. The user specifies a target defocus for tuning and may optionally request the insertion of an objective aperture. Tuning can be performed either at the predefined ’sample’ position or at the current stage position, which is useful if only the tuning part of the workflow is required. Before the automated procedure starts, the user can refine the FOV to ensure that the power spectrum contains sufficient Thon-ring signal for automated astigmatism and coma-free alignments by CTF as implemented in *SerialEM*. The desired defocus is then set through stage-height adjustment and, if previously inserted, the objective aperture is retracted. The automated part of the routine first corrects objective-lens astigmatism and then performs coma-free alignment. If requested, the objective aperture is subsequently inserted and manually centred, followed by a final correction of aperture-induced astigmatism. When the tuning routine is skipped, the ‘aperture routine’ for objective aperture insertion and centring can be executed independently. This grouping of calibration and tuning functions allows the workflow to accommodate both complete setup sessions and partial reconfiguration of an already prepared microscope.

### 2.4. Software architecture and configurability

*LowDoseWizard* is implemented entirely through the native scripting interface of *SerialEM* and does not require additional software beyond *SerialEM* itself. It uses only native script commands and is compatible with *SerialEM* version 4.2.2 or higher, although version 4.3.0beta or later provides the best user experience. The software architecture is deliberately modular and is divided into configuration files, scripts and reusable functions. Only the configuration files need to be adapted for a specific microscope; the scripts and functions themselves remain unchanged. This separation allows the workflow logic to be maintained independently of instrument-specific parameter definitions and facilitates transfer of the workflow between different microscopes.

Three plain-text configuration files define the microscope-specific parameters used by the workflow (Table 1 ▸). The system configuration file (wizard_system_config.txt) specifies general instrument and workflow settings, such as the presence of an energy filter, the use of a two- or three-condenser system and the data-acquisition workflows. The optics configuration file (wizard_optics_config.txt) contains microscope-specific definitions for imaging conditions, including magnifications, spot sizes, beam settings and aperture choices. The camera configuration file (wizard_camera_config.txt) defines detector-related settings such as fluence, exposure time and binning. These files are intended to be prepared once by expert staff or the microscope manager.

At the script level, *LowDoseWizard* consists of one master script and six routine scripts (Table 1 ▸). Only the master script is called directly by the user; it checks the required pre­requisites in the *Navigator* and then invokes the selected routines in sequence (Fig. 1 ▸). Each routine may be skipped, and the full workflow may be terminated between routines if necessary. In addition, frequently reused subroutines are implemented as *SerialEM Functions*. In the current implementation, eight functions are used, including functions for low-dose area setup, camera setup, determination of eucentric height by focus and interactive stage-position refinement (Eisenstein *et al.*, 2023 ▸). Because these functions are modular, they can also be called from other *SerialEM* scripts outside *LowDoseWizard*. Overall, this architecture was designed to preserve the flexibility of *SerialEM* while providing a standardized, guided front-end for routine cryo-TEM setup.

### 2.5. Output files and session reuse

Once the optics and camera routines have been completed, *LowDoseWizard* automatically generates a summary file containing the critical microscope and imaging parameters of the session and saves it in the *SerialEM* project folder (Supplementary Video S1). In addition to this human-readable summary, the workflow creates a session parameter file that can be used to initialize subsequent imaging sessions on the same microscope with the same parameter set. When such a parameter file is reused, the initial prompts of the optics and camera routines can be skipped. This output structure provides both a record of the configured imaging conditions and a practical mechanism for reusing validated parameter combinations across repeated sessions.

## 3. Results

### 3.1. *LowDoseWizard* enables rapid routine cryo-TEM setup

**Figure 3:**
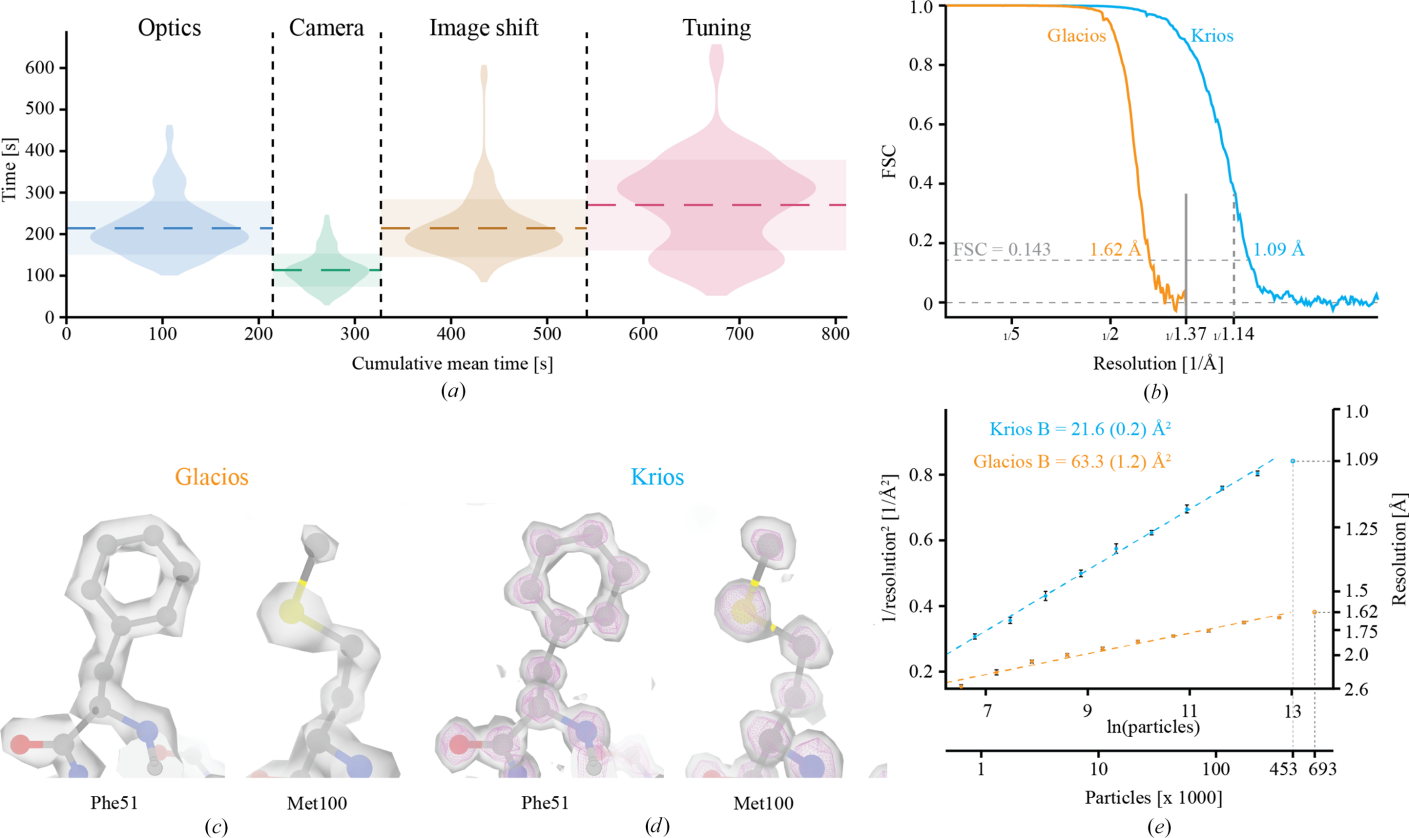
(*a*) Runtime of *LowDoseWizard*. Dashed horizontal lines represent the mean runtime of each routine. Horizontal transparent bars represent the respective standard deviation and violin plots represent the raw data (optics, *n* = 67; camera, *n* = 65; image shift, *n* = 67; tuning, *n* = 45). (*b*) Gold-standard FSC plot of half-maps from the Krios (cyan) and Glacios (orange) data sets. The grey vertical line indicates the Nyquist frequency of the Glacios data set. The dashed vertical line indicates the physical Nyquist frequency of the Krios data set. FSCs were calculated with *relion_postprocess*. (*c*) Densities from the Glacios data (level 0.2). (*d*) Densities from the Krios data of same residues as in (*c*) shown at low (grey surface, level 0.2) and high (magenta mesh, level 0.52) thresholds. PDB entry 7a4m was fitted into all densities. All densities were generated with *relion_postprocess*. (*e*) Rosenthal–Henderson *B*-factor plot (Rosenthal & Henderson, 2003 ▸) of the Krios (cyan) and Glacios (orange) data sets. Error bars represent the standard deviation of the resolution estimation from the random resampling and the number in parentheses represents the standard deviation of the fitted *B* factor. The respective data point of the full particle stack (not included in the straight-line fit) is highlighted by dashed lines.

To assess the practical runtime of the workflow under routine operating conditions, *LowDoseWizard* automatically logged the duration of individual routines during user sessions on three cryo-TEMs at EMBL Heidelberg, comprising one Glacios and two Titan Krios microscopes. Timings were collected across sessions performed by 13 users, thereby capturing typical variation arising from both user interaction and microscope-specific implementation. Across all measurements, the mean cumulative runtime of the workflow was 13.5 min (Fig. 3 ▸*a*). Among the individual routines, the camera routine was the fastest and showed the smallest standard deviation, whereas the tuning routine was the slowest and displayed the largest variability (Table 2 ▸).

The limited variation in runtime of the camera routine is consistent with the small amount of user input required during this part of the workflow, which is largely determined by automated flux calibration and assignment of exposure conditions. By contrast, the larger spread observed for the tuning routine likely reflects the dependence of astigmatism correction and coma-free alignment on the availability of suitable image features and Thon-ring signal in the chosen field of view. The image-shift routine similarly showed greater variability, consistent with the need for interactive refinement of stage position before calibration. Taken together, these measurements indicate that *LowDoseWizard* supports rapid microscope preparation in routine use, with total setup typically completed within approximately 15 min while retaining the flexibility to accommodate user- and sample-dependent adjustment during calibration and tuning.

### 3.2. Validation on Glacios and Titan Krios microscopes

To test whether the use of predefined imaging conditions generated by *LowDoseWizard* is compatible with high-quality cryo-EM data acquisition, we applied the workflow to benchmark SPA data collection on two microscopes at the EMBL Imaging Centre: a Glacios (Thermo Fisher Scientific) operated at 200 kV and a Titan Krios G4 (Thermo Fisher Scientific) operated at 300 kV. The Glacios was equipped with an X-FEG source, fringe-free imaging and a 20 µm C2 aperture (Konings *et al.*, 2019 ▸), whereas the Krios was equipped with an E-CFEG source. Both microscopes were fitted with a Selectris X energy filter and a Falcon 4i direct electron detector.

The validation was designed to reflect realistic routine operating conditions. After loading the respective alignment file at the start of the session, no additional daily alignments were performed beyond those covered by *LowDoseWizard* itself. In practice, this meant that beam-shift calibration, image-shift offset calibration, objective-lens astigmatism correction and coma-free alignment were performed within the workflow. Of note, both TEM alignment files were last modified after major service interventions about one year prior to data collection. In addition, before starting automated data acquisition, *Coma versus Image Shift* was calibrated in *SerialEM* and energy-filter tuning was carried out, including zero-loss peak centring, isochromatic tuning and distortion tuning. This setup allowed assessment of whether a workflow based on predefined, microscope-specific parameter sets, supplemented by a limited number of essential session-specific calibration steps, was sufficient to support high-resolution single-particle data acquisition on both a two-condenser and a three-condenser microscope.

### 3.3. Benchmark single-particle data acquisition and reconstruction

Mouse heavy-chain apoferritin was used as a benchmark specimen for validation of the imaging conditions produced by *LowDoseWizard*. The sample was prepared at 3.2 mg ml^−1^ in 20 m*M* HEPES pH 7.5, 80 m*M* NaCl, 1.7 m*M* DTT. Aliquots of 3 µl were applied to glow-discharged Quantifoil R2/1 Cu 300 mesh grids carrying an additional 2 nm carbon support film. After a waiting time of 15 s, grids were blotted for 1.5 s from the sample side using a Leica EM GP2 operated at 8°C and 99% relative humidity and were plunge-frozen in liquid ethane. Data acquisition on the Titan Krios and Glacios microscopes was performed from the same grid. The principal data-collection parameters are summarized in Table 3 ▸.

For the Glacios dataset, movie processing was carried out in *cryoSPARC* v.5.0.0 (Punjani *et al.*, 2017 ▸). Movies were imported as 20 exposure groups spanning approximately 30 min intervals. After patch motion correction and patch CTF estimation, exposures with estimated astigmatism greater than 150 Å or estimated CTF resolution worse than 3 Å were discarded, leaving 2723 micrographs for further processing. Particles were picked with *crYOLO* using the general pretrained model (Wagner *et al.*, 2019 ▸), yielding 750 421 extracted particles. After one round of 2D classification to remove poorly resolved particles and contaminants, 693 767 particles were retained. Refinement with octahedral symmetry, including per-particle defocus refinement, beam-tilt correction, higher-order aberration refinement and Ewald-sphere correction, yielded a reconstruction at 1.72 Å resolution. One round of reference-based motion correction further improved the final masked resolution to 1.62 Å (Figs. 3 ▸*b* and 3 ▸*c*).

For the Titan Krios dataset, movies were imported into *RELION* 5.0 (Kimanius *et al.*, 2024 ▸) and motion-corrected using *RELION*’s own implementation. CTF estimation was performed with *CTFFIND*-4.1 (Rohou & Grigorieff, 2015 ▸). After the exclusion of micrographs with estimated CTF fit worse than 3 Å, astigmatism greater than 100 Å, defocus higher than −1.2 µm or CTF figure of merit below 0.2, 2839 micrographs were retained. Particles were picked with *crYOLO* and classified (2D) initially in *cryoSPARC* v.4.7.1. After the removal of poorly resolved classes, 453 035 particles were retained for refinement. Subsequent refinement with octahedral symmetry, per-particle defocus refinement, beam-tilt correction, higher-order aberration refinement and Ewald-sphere correction yielded a reconstruction at 1.21 Å resolution. Bayesian polishing in *RELION* followed by further homogeneous refinement in *cryoSPARC* improved the final masked resolution to 1.15 Å, and subdivision of the dataset into six optics groups spanning approximately 60 min intervals further improved the final resolution to 1.09 Å (Figs. 3 ▸*b* and 3 ▸*d*).

The benchmark reconstructions obtained from the two datasets show that the imaging conditions generated by *LowDoseWizard* are compatible with high-resolution SPA cryo-EM on both microscope classes tested. The Glacios dataset yielded a final masked reconstruction at 1.62 Å resolution, whereas the Titan Krios dataset yielded a final masked reconstruction at 1.09 Å resolution (Table 3 ▸; Figs. 3 ▸*b*, 3 ▸*c* and 3 ▸*d*). These results indicate that rapid setup based on pre­defined, microscope-specific acquisition parameters does not preclude the collection of data suitable for near-atomic and atomic-resolution structure determination.

To assess data quality further, Rosenthal–Henderson *B* factors were estimated from repeated random subsampling of the final particle stacks followed by homogeneous refinement in *cryoSPARC* (Nakane *et al.*, 2020 ▸). The resulting *B* factors were 63.3 Å^2^ for the Glacios dataset and 21.6 Å^2^ for the Titan Krios dataset (Fig. 3 ▸*e*). These values are consistent with the high overall quality of both datasets and support the conclusion that on modern cryo-TEMs, predefined imaging conditions combined with a limited number of guided calibration and tuning steps can provide acquisition conditions suitable for high-resolution SPA. Of note, high resolutions and low *B* factors could be achieved despite the added noise from the carbon film. Under the conditions tested here, this applied to both a 200 kV microscope equipped with fringe-free imaging and a 300 kV microscope equipped with an energy filter and E-CFEG source.

## 4. Conclusions

*LowDoseWizard* addresses a practical limitation of routine cryo-TEM operation by reducing the setup of low-dose imaging conditions to a guided and standardized workflow implemented in *SerialEM*. The workflow translates a small number of user-defined choices into microscope optics and camera settings, and combines these with the calibration and tuning steps required for reliable acquisition. In routine use across multiple users and microscopes, complete setup was achieved in approximately 15 min. Validation on a 200 kV Glacios and a 300 kV Titan Krios further showed that the predefined, microscope-specific parameter sets used by the workflow are compatible with high-resolution single-particle cryo-EM data acquisition, yielding apoferritin reconstructions at 1.62 and 1.09 Å resolution, respectively. Together, these results show that rapid and guided cryo-TEM setup does not compromise the quality of the resulting data under the conditions tested here.

The principal value of *LowDoseWizard* lies in combining the flexibility of *SerialEM* with a more accessible and reproducible setup procedure. Commercial acquisition environments provide strongly guided workflows for standard operation, but open platforms such as *SerialEM* remain important where imaging conditions need to be adapted to specific specimens or where new acquisition strategies are introduced. By structuring key setup decisions through editable configuration files and modular routines, *LowDoseWizard* preserves this flexibility while reducing the burden of routine parameter selection and daily alignment. This should be particularly useful in shared instrumentation environments, where users vary in experience and where microscope time is best devoted to specimen-specific tasks such as target selection. The same considerations are likely to be especially relevant for cryo-ET and correlative workflows, in which efficient setup of robust low-dose imaging conditions is only one part of a broader and often complex acquisition strategy (Nogales & Mahamid, 2024 ▸, Young & Villa, 2023 ▸).

## Supplementary Material

EMDB reference: mouse heavy-chain apoferritin on continuous carbon from 300 kV Titan Krios, EMD-57470

EMDB reference: from 200 kV Glacios, EMD-57471

Screen capture of the LowDoseWizard workflow on a Glacios microscope. DOI: 10.1107/S2059798326006327/sor5006sup1.mp4

## Figures and Tables

**Table 1:** *LowDoseWizard* files

Script Name	File Name	Purpose
*LowDoseWizard*	lowdosewizard.txt	Master script: calls all routines
*wizard_optics*	01_wizard_optics.txt	Optics routine: sets magnifications and beam parameters
*wizard_camera*	02_wizard_camera.txt	Camera routine: calibrates flux and sets exposure times
*wizard_imageshift*	03_wizard_imageshift.txt	Image-shift routine: aligns FOV across all low-dose states
*wizard_tune*	04_wizard_tune.txt	Tuning routine: automated astigmatism correction and coma-free alignment
*wizard_objap*	05_wizard_objap.txt	Objective aperture routine: inserts objective aperture and guides centring
*wizard_summary*	06_wizard_summary.txt	Summary routine: generates summary file
Functions	functions.txt	Required *SerialEM* functions
n.a	wizard_system_config.txt	Configuration file for general microscope parameters
n.a.	wizard_optics_config.txt	Configuration file for microscope optics definitions
n.a.	wizard_camera_config.txt	Configuration file for camera parameter definitions

**Table 2:** *LowDoseWizard* routine runtime SD, standard deviation; *n*, number of measurements

Routine	Mean (s)	SD (s)	*n*
Optics	214	64	67
Camera	113	40	65
Image shift	214	69	67
Tuning	270	109	45

**Table 3:** Cryo-EM data collection and reconstruction

Microscope	Glacios 1	Titan Krios G4
Acceleration voltage (kV)	200	300
Mode	Nanoprobe EFTEM	Nanoprobe EFTEM
Magnification (nominal)	165000	215000
Calibrated pixel size (Å)	0.686	0.572
C2 aperture size (µm)	20	50
Objective aperture (µm)	Retracted	Retracted
Beam size (nm)	650	420
Energy filter slit width (eV)	10	10
Exposure time (s)	2	1.3
Fluence (e^−^ Å^−2^)	40	40
EER fractions	648	414
Exposures per foil hole	9	17
Acquired foil holes per stage movement	1	1
Automation software	*SerialEM* 4.2.0beta	*SerialEM* 4.2.0beta
No. of movies collected	3033	3417
No. of movies used	2723	2839
No. of particles extracted	750421	593971
Final No. of particles	693767	453035
Point-group symmetry	*O*	*O*
Resolution (global) (Å)		
FSC 0.143 (unmasked/masked)	1.82/1.62	1.27/1.09
Map-sharpening *B* factor (Å^2^)	−37	−15

## Data Availability

All LowDoseWizard files including documentation are available on the EMBL Git (https://git.embl.org/grp-ic/lowdosewizard). Apoferritin reconstructions are available as EMDB entries EMD-57470 (Titan Krios) and EMD-57471 (Glacios). The raw EER data have been deposited as EMPIAR entries EMPIAR-13508 (Titan Krios) and EMPIAR-13509 (Glacios).
